# Development of a novel patient-reported outcome measure for disseminated coccidioidomycosis (valley fever)

**DOI:** 10.1093/jac/dkae453

**Published:** 2024-12-17

**Authors:** Emma Harvey, Jennifer Clegg, Mark Bresnik, Elliott Blatt, Sophie Hughes, Cindy Umanzor-Figueroa, Rob Purdie, George R Thompson, Tara Symonds

**Affiliations:** Clinical R&D, F2G, Ltd, Manchester, UK; Clincial Outcomes Assessments, Clinical Outcomes Solutions, Chicago, IL, USA; Clinical R&D, F2G, Ltd, Manchester, UK; Clincial Outcomes Assessments, Clinical Outcomes Solutions, Chicago, IL, USA; Clinical Outcomes Assesments, Clinical Outcomes Solutions, Folkestone, Kent, UK; Clincial Outcomes Assessments, Clinical Outcomes Solutions, Chicago, IL, USA; Patient Coordination, Valley Fever Institute at Kern Medical, Bakersfield, CA, USA; MyCology Advocacy, Research and Education (MyCARE), Colorado Springs, CO, USA; Division of Infectious Diseases, Department of Internal Medicine, UC Davis Health, Sacramento, CA, USA; Clinical Outcomes Assesments, Clinical Outcomes Solutions, Folkestone, Kent, UK

## Abstract

**Background:**

Coccidioidomycosis (Valley Fever) is a dimorphic fungal infection endemic to the southwest United States, Mexico, Central and South America, which can lead to chronic debilitating illness and death.

**Objectives:**

This qualitative study was conducted to develop a bespoke patient-reported outcome measure for patients with chronic disseminated coccidioidomycosis to assess health-related quality of life (HRQoL) impacts.

**Patients and methods:**

Online, first-person narratives of patient experiences of disseminated coccidioidomycosis were used to create a patient-centred conceptual model of symptoms and impacts of the condition. Interviews were conducted with expert clinicians, followed by concept elicitation interviews with patients, to generate key symptom and impact concepts from which an initial patient-reported outcome measure was developed. The draft measure was reviewed with clinicians for clinical relevance and further assessed in cognitive debriefing interviews with patients to refine the measure and establish content validity.

**Results:**

A total of 25 patients were interviewed, which identified impacts relating to physical function, activities of daily living, cognitive function, social/role function and emotional function of disseminated coccidioidomycosis. Twenty items were developed simultaneously in English and Spanish to capture the main impacts across these 5 areas. In general, items were clear and well understood by patients, and clinicians found the measure clinically relevant and appropriate for assessing the burden of disseminated coccidioidomycosis on HRQoL.

**Conclusions:**

Once fully validated, the Valley Fever—Patient Reported Outcome measure may be used in interventional studies to assess HRQoL outcomes, and in clinical practice to monitor patients’ HRQoL.

## Introduction

Coccidioidomycosis [Valley Fever (VF)] is a fungal disease acquired by inhaling arthroconidia of *Coccidioides* spp., a dimorphic fungus endemic to southwest USA and parts of Mexico and Central/South America. Infection follows inhalation into the lungs and can disseminate to other parts of the body, leading to chronic debilitating illness and death, particularly if the CNS is involved. Coccidioidomycosis is increasingly prevalent and widening in geographical distribution, with climate change being a contributing factor.^1,2^ In 2019, there was an estimated incidence of 20 000 diagnosed cases of VF across the southwestern USA, although it is likely this is under reported as many patients will have a self-limiting infection, which is difficult to differentiate from other forms of respiratory illness or community acquired pneumonia.^3^ Reported cases are known to grossly underrepresent actual case counts due to the high percentage of patients experiencing asymptomatic or sub-clinical infection.^4^ An estimated 1%–3% of cases (∼2000 per year) progress to some form of disseminated coccidioidomycosis.^5–7^ Patients with disseminated infections require close follow-up and long periods of antifungal therapy in an attempt to reduce the high morbidity and/or mortality in those with severe infections.

Assessing outcomes and response to antifungal therapy in coccidioidomycosis can be challenging because clinical signs and symptoms vary widely between patients and some manifestations may persist even after control of the underlying infection (e.g. fatigue). Serology is helpful for both diagnosis and to monitor the response to therapy. Antigen testing has not been evaluated for longitudinal use and is generally useful only in profoundly immunosuppressed patients for diagnosis due to its limited sensitivity in immunocompetent hosts. In patients with CNS infection, measuring the cell count, protein and glucose in the cerebrospinal fluid can be useful but may not follow a clear course of improvement, complicating follow-up.

Measuring patients’ health-related quality of life (HRQoL) is increasingly recognized as an important component of clinical trial outcomes and within clinical care.^8^ As current clinical trial assessments may not accurately reflect how patients feel or function, there is a growing need for patient-reported outcome (PRO) instruments that measure the impact of the condition on patients’ quality of life and ability to perform daily activities, particularly given the chronic, debilitating nature of disseminated coccidioidomycosis.

Currently, there are no PRO instruments that assess the patients’ experience of symptoms or impacts of coccidioidomycosis. In preliminary work, a literature review found generic PROs, such as the PROMIS-10, lacked the specificity needed to measure the HRQoL impacts of disseminated coccidioidomycosis.^9^ Further, a recent olorofim study demonstrated zero overall response rates when using the EORTC/MSG criteria, which combines clinical, radiological and mycological measures.^10,11^ This lack of response is due to the persistence of serologic positivity regardless of symptomatic improvement, rather than therapeutic failure in coccidioidomycosis. These serologic tests may improve over time; however, any remaining positive result is adjudicated, for clinical trial purposes, as mycologic persistence (and therefore treatment failure)—a limitation of the current EORTC/MSG criteria when applied to coccidioidomycosis and a reminder they were developed for non-coccidioidal fungal diseases. The observed clinical and/or radiographic improvement is negated on the basis of positive serologic testing, reinforcing the need to develop a disease-specific PRO instrument. The goal of this research was to develop a comprehensive, novel PRO measure assessing the HRQoL impacts on patients with disseminated coccidioidomycosis.

## Materials and methods

### Study design

A targeted literature review was conducted to determine whether any PRO measures assessing HRQoL impacts, particularly functional impacts, had been developed and validated for patients with any type of invasive fungal infection. There were no measures identified across Ovid and PROQOLID database searches. A literature review was conducted to identify papers on the patient experience of coccidioidomycosis to aid in the development of an initial draft of a conceptual model (CM) from the patient perspective. However, this search also yielded no results.

Therefore, the ‘grey literature’ was utilized to generate a conceptualization of the disease from the patient’s perspective. For this search, a data source was defined as ‘an open access website where information is presented, posted and/or discussed relating to the target topics and subject areas’. Largely, VF awareness or advocacy articles in the lay press and/or websites with first person narratives about the patient experience of the condition were utilized. Sites requiring membership or login were excluded. Qualitative data analysis of the information found led to the development of a preliminary CM of coccidioidomycosis-related symptoms and impacts.

**Figure 1.**
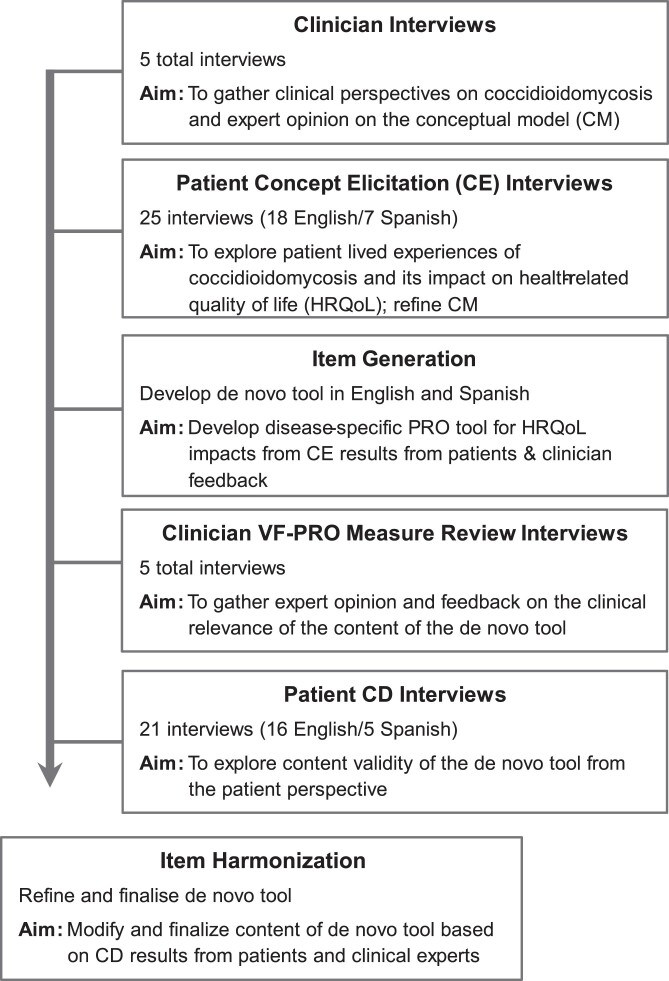
Overview of study design.

The remainder of the study was conducted in 2 phases designed to incorporate the steps of measure development for a *de novo* PRO instrument (Figure 1).^8,12^ Phase 1 began with interviews with expert clinicians to gather clinical insights on patient subgroups of disseminated coccidioidomycosis and the draft CM. Patient concept elicitation (Ce) interviews followed to understand the lived experience of the condition and identify key symptom and impact concepts. These concepts were used to revise the draft CM and generate items for the PRO measure with a focus on the HRQoL impacts on patients’ lives.

Phase 2 of the study consisted of cognitive debriefing (CD) interviews with patients to evaluate the content validity and comprehensiveness of the initial draft measure. The results of the CD interviews were used to revise and refine the draft PRO measure. The updated measure was then reviewed by clinicians for feedback on clinical relevance, item difficulty level and any missing concepts.

No medical procedures, therapeutic interventions or treatments were administered as part of this study. Study exempt status was granted from the WCG Independent Review Board^®^ on June 28, 2021. This study was conducted in compliance with Good Clinical Practice guidelines; all applicable local laws and regulatory requirements were adhered to throughout the study.^13^

### Study participants

Patient participants in this observational study were identified and recruited using 2 methods: (i) direct patient referrals from a patient advocate and (ii) patient outreach at one US-based site (Valley Fever Institute, Bakersfield, CA, USA). Eligible patients were required to have a clinician-confirmed diagnosis of chronic and disseminated coccidioidomycosis. ‘Chronic’ patients were defined in the eligibility criteria as those with inadequately controlled symptoms attributable to their VF infection while on antifungal treatment for >6 months and had failed at least one azole therapy, a marker of disease severity. Participants were excluded if they had a comorbid condition in which symptoms may overlap with disseminated coccidioidomycosis symptoms. Clinician participants were MDs with a specialty in infectious disease and ≥5 years of experience in the diagnosis, treatment and management of patients with disseminated coccidioidomycosis.

Recruitment targets for the patient Ce and CD interviews were established to ensure a diverse group in terms of age, race, educational level, geographical area of infection and dissemination type. Patient participants provided informed consent prior to enrolment and received an honorarium after completing a Ce or CD interview. Clinicians received compensation for their time at market-rate value, and a patient advocate was compensated as a consultant on the project.

### Qualitative interviews

Interviews were conducted in English or Spanish via telephone or teleconference software. Semistructured interview guides were developed for both patient and clinician interviews. The interview guides featured open-ended questions to facilitate and encourage spontaneous, participant-led discussion of topics and support the elicitation of symptom and impact concepts.

The purpose of the interviews with clinicians was to gather expert clinical opinion on the key symptoms of coccidioidomycosis and impact on patients’ HRQoL. Clinicians were also asked to provide feedback on distinct patient subgroups of disseminated coccidioidomycosis (CNS meningitis, bone/joints, skin/cutaneous and multi-site dissemination) and on the accuracy of the draft CM based on their clinical experience treating these patients. The patient Ce interviews explored the lived experience of disseminated coccidioidomycosis with a focus on patients’ descriptions of how coccidioidomycosis impacted their lives.

Following analysis, the draft CM was updated with key symptom and impact concepts based on frequency of mention and overall significance described by patients. Key insights from clinicians and a patient advocate were integrated into the revisions, with a focus on retaining concepts that were found in disseminated coccidioidomycosis and removing those typically found in primary pulmonary disease.

PRO items were drafted based on key concepts found in the CM, i.e. most important HRQoL impacts from the patient perspective. Patients’ words, terminology or phrasing were used in item development whenever possible. Response options and recall period were selected based on patients’ experiences of the concept alongside PRO regulatory guidance.^12^

The patient CD interviews were conducted to evaluate the content validity and ensure clarity, relevance and comprehensiveness of the PRO measure. Clinician interviews were not a formal debriefing of the measure, but rather a ‘measure review’ used to gather clinicians’ feedback on the newly developed PRO instrument, focusing on the clinical relevance and difficulty level of the concepts assessed by the new measure.

### Analysis

Interviews were audio-recorded and transcribed. The transcribed data were analysed using NVivo 1.6.1, a software package that is designed to facilitate the coding and systematic analysis of qualitative data. Coding and analyses were completed by trained researchers.^14^

Interview transcripts were analysed using a thematic analysis approach in which the researcher identifies, analyses and interprets patterns of meaningful text, or ‘themes’, related to the research objectives.^15^ This approach allows researchers to be reflective to generate a broad picture of the patient experience of the symptoms and impacts of disseminated coccidioidomycosis.

The FDA recognizes concept saturation as ‘the point when no new relevant or important information emerges and collecting additional data will not likely add to the understanding of how patients perceive the concept of interest^’^.^8,16–18^ Saturation analysis was conducted on the patient Ce interview transcripts by comparing language and CNS subgroups (English and Spanish-speaking patients, and CNS and non-CNS patients); these analyses also functioned to highlight differences in subgroup experiences.

CD data were analysed using a structured, deductive approach that systematically compared participant responses to questions about the understanding, relevance, suitability and feasibility of the instructions, item concepts, response scale/options and recall period of the PRO measure.

## Results

### Participant characteristics

Five clinicians took part in the initial interviews discussing key symptom and impacts, and thoughts on the draft CM, 3 of whom also participated in the review of the proposed content for the draft PRO measure alongside 2 new clinicians. All clinicians were infectious disease specialists with ≥7 years of experience, with a mean of 22.6 and 20.2 years for the first clinician interview cohort and second interview cohort, respectively.

With respect to the patient-participant Ce sample, 52.0% were White and 48.0% were male. The median age of patients was 49.2 years (23.5–71.4 years). Nearly one-half of the patients (48.0%) were of Hispanic descent. The median time since being diagnosed with disseminated coccidioidomycosis was 4.7 years (0.5–32.7 years). Half of the patients had CNS involvement (52.0%); the most common non-CNS sites were bone/joint/musculoskeletal (36.0%) and persistent pulmonary disease (36.0%). Patients most frequently reported their current disease activity as moderate (44.0%).

Patient demographic and clinical characteristics were similar for the CD sample, given it was a subset of the Ce patient interview population. One notable difference was that milder current disease activity was reported at the time of the CD interviews, which occurred ∼1 year after the Ce interviews. Ce interviews were conducted from November 2021 to October 2022 and the CD interviews from June 2023 to July 2023.

Demographic and clinical characteristics for the patient-participant sample is shown in Table 1.

**Table 1.** Demographic and clinical characteristics of Ce and CD interview samples

Variables		Ce interview total (*N* = 25; English, *n* = 18; Spanish, *n* = 7)	CD interview total (*N* = 21; English, *n* = 15; Spanish, *n* = 6)
Gender, *n* (%)	Male	12 (48.0%)	11 (52.4%)
	Female	13 (52.0%)	10 (47.6%)
Age	Mean (SD)	47.29 (12.8)	48.49 (12.7)
	Median	49.17	50.24
Ethnicity	Hispanic/Latino	12 (48.0%)	11 (52.4%)
	Not Hispanic/Latino	13 (52.0%)	10 (47.6%)
Race^a^	White/Caucasian	13 (52.0%)	9 (42.9%)
	Black/African American	3 (12.0%)	3 (14.3%)
	Latin American, Mexican, Cuban or of Spanish descent	6 (24.0%)	6 (28.6%)
	Other	6 (24.0%)	5 (23.8%)
Education	Did not complete high school	4 (16.0%)	3 (14.3%)
	High school diploma or General Education Diploma	2 (8.0%)	2 (9.5%)
	Some college or certification programme	7 (28.0%)	4 (19.0%)
	College, technical college or university degree (2- or 4- year)	8 (32.0%)	8 (38.1%)
	Graduate degree (MS, PhD, MD, etc.)	2 (8.0%)	2 (9.5%)
	Other	2 (8.0%)	2 (9.5%)
	*n* missing	0	0
Work status^a^	Employed/self-employed full-time	10 (40.0%)	7 (33.3%)
	Employed/self-employed part-time	3 (12.0%)	3 (14.3%)
	Homemaker	1 (4.0%)	0
	Disabled	7 (28.0%)	7 (33.3%)
	Current student	2 (8.0%)	1 (4.8%)
	Retired	2 (8.0%)	2 (9.5%)
	Unemployed	4 (16.0%)	4 (19.0%)
Time since diagnosis (years)	Mean	7.17 (7.409)	6.96 (7.768)
	Median	4.72	4.72
Area of dissemination or infection^a^	CNS/meninges	13 (52.0%)	10 (47.6%)
	Skin/cutaneous	4 (16.0%)	4 (19.0%)
	Bone/joint/musculo-skeletal	9 (36.0%)	9 (42.9%)
	Pulmonary/lungs	9 (36.0%)	9 (42.9%)
	Lymphatic system (lymph nodes)	1 (4.0%)	1 (4.8%)
	Other	0	3 (14.3%)
Current disease activity^b^	Very mild	0	2 (9.5%)
	Mild	5 (20.0%)	7 (33.3%)
	Moderate	11 (44.0%)	4 (19.0%)
	Severe	6 (24.0%)	2 (9.5%)
	Very severe	3 (12.0%)	3 (14.3%)
	*n* missing	0	3 (14.3%)
Location participant acquired infection	Arizona	6 (30.0%)	5 (29.4%)
	California	12 (60.0%)	10 (58.8%)
	Texas	0	0
	Other state or ‘don’t know’	2 (10.0%)	2 (11.8%)
	*n* missing	5	4
Current treatment^a^	Fluconazole	12 (48.0%)	12 (57.1%)
	Voriconazole	0	0
	Isavuconazole	4 (16.0%)	2 (9.5%)
	Itraconazole	2 (8.0%)	1 (4.8%)
	Posaconazole	6 (24.0%)	5 (23.8%)
	IV liposomal amphotericin B or other formulation	0	0
	Intrathecal drugs	2 (8.0%)	0
	Other	1 (4.0%)	1 (4.8%)
Previous treatment^a^	Fluconazole	13 (52.0%)	9 (42.9%)
	Voriconazole	8 (32.0%)	5 (23.8%)
	Isavuconazole	0	0
	Itraconazole	4 (16.0%)	3 (14.3%)
	Posaconazole	4 (16.0%)	4 (19.0%)
	IV liposomal amphotericin B or other formulation	7 (28.0%)	5 (23.8%)
	Intrathecal drugs	1 (4.0%)	1 (4.8%)
	Other	0	0

CD, cognitive debrief; Ce, concept elicitation; SD, standard deviation.

^a^Respondents were asked to select all categories that applied to their situation.

^b^Current disease severity reflects both patient-reported and clinician-reported severity based on the recruitment source.

### Clinician feedback

**Figure 2.**
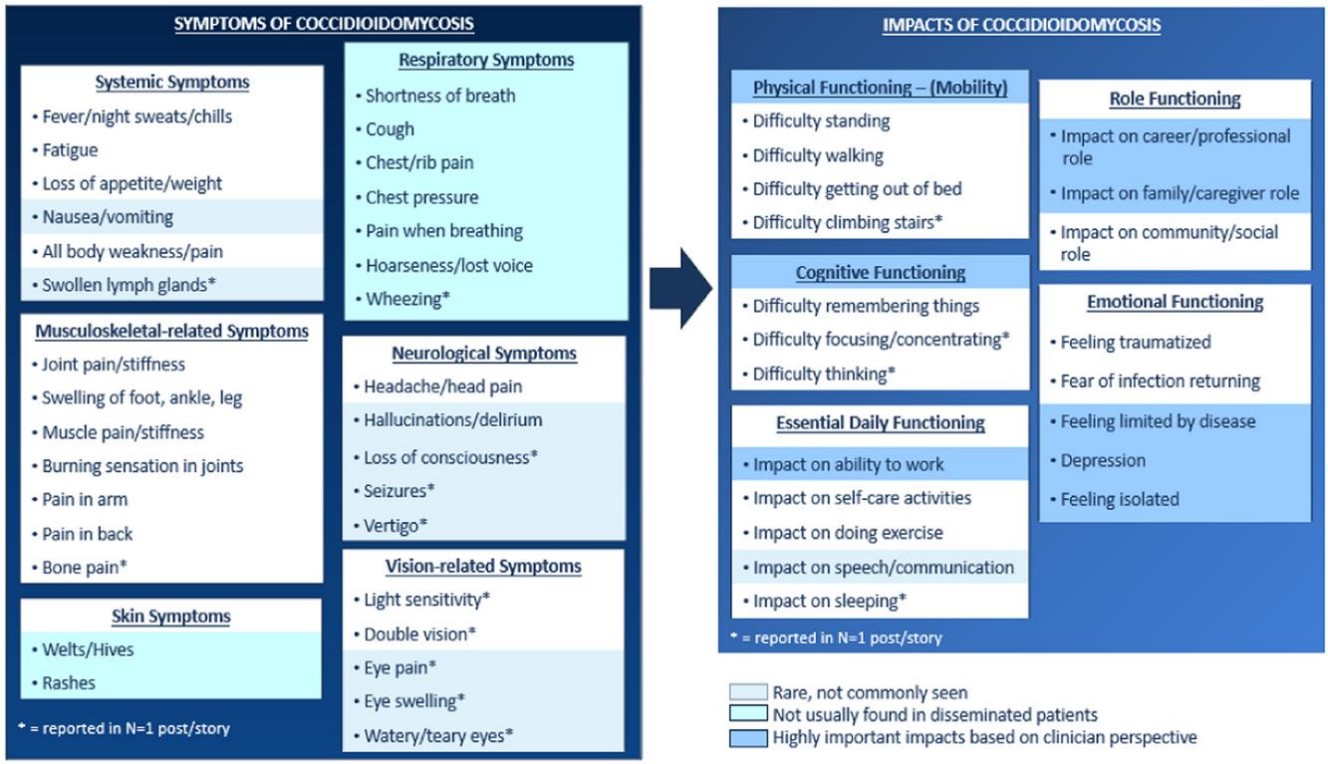
Clinician feedback on preliminary conceptual model of coccidioidomycosis.

Clinicians provided insightful feedback on symptoms experienced with disseminated coccidioidomycosis and identified areas in the CM that were more typical of primary pulmonary disease and should be removed. The majority considered difficulties with physical functioning (e.g. mobility) and work as most important impacts to their patients, followed by cognitive impairments and depression/isolation (Figure 2).

### Patient CE findings

The Ce interviews (*N* = 25) provided rich, qualitative data on English-speaking (*n* = 18) and Spanish-speaking (*n* = 7), and CNS (*n* = 13) and non-CNS (*n* = 12) patient experience of disseminated coccidioidomycosis. The most frequently experienced symptom concepts reported by patients were fatigue (*n* = 25), headache (*n* = 25), fever/night sweats/chills (*n* = 23), musculoskeletal pain (*n* = 23) and shortness of breath (*n* = 23). The most frequently reported impacts were difficulty doing physical activity/exercise (*n* = 25), impact on work/school (*n* = 24), impact on sleep (*n* = 24) and impact on walking (*n* = 23). In total, >200 distinct symptom and impact concepts were reported by patients; thus, it should be noted that similar symptoms or impacts concepts were consolidated into broader concepts; for example, ‘musculoskeletal pain’ consists of joint, muscle and bone pain.

The Ce interviews revealed that there were no major differences between the CNS and non-CNS patient experience. Notable minor differences included some respiratory symptoms being experienced more frequently in non-CNS patients, and only CNS patients discussed neurological symptoms such as head pressure, stroke-related symptoms and paralysis. Additionally, ataxia, described by patients as balance issues, instability or falling, was more frequently experienced in CNS patients (46% versus 17%). Most impact concepts were discussed across the 2 groups; however, only CNS patients reported impacts on sexual function (*n* = 3) and more frequently experienced difficulty speaking/communicating (92% versus 67%).

**Figure 3.**
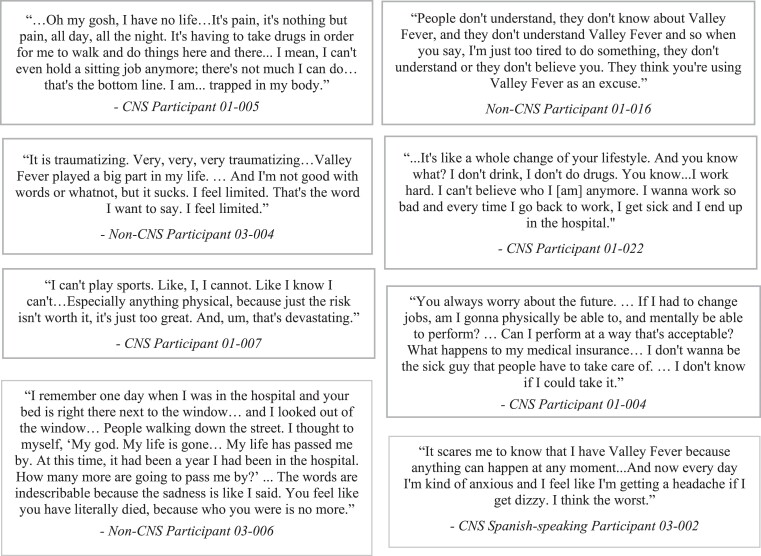
Patient quotes describing the HRQoL impacts of disseminated coccidioidomycosis.

Symptom experiences were similar between English- and Spanish-speaking patients; however, gastrointestinal symptoms, certain musculoskeletal (muscle strain, numbness, weakness in extremities) and neurological symptoms [balance/instability/falling (ataxia), light-headedness, nerve-related issues] were not reported by Spanish-speaking patients. In terms of impact concepts, only English-speaking patients reported impacts on career/professional role (*n* = 17), feeling judged/stigmatised/lack of understanding (*n* = 12), feeling angry/frustrated/irritable (*n* = 8), impact on eating/drinking (*n* = 5) and feeling like a burden to others (*n* = 5). Patient quotes are provided to illustrate the HRQoL impacts of disseminated coccidioidomycosis (Figure 3).

**Figure 4.**
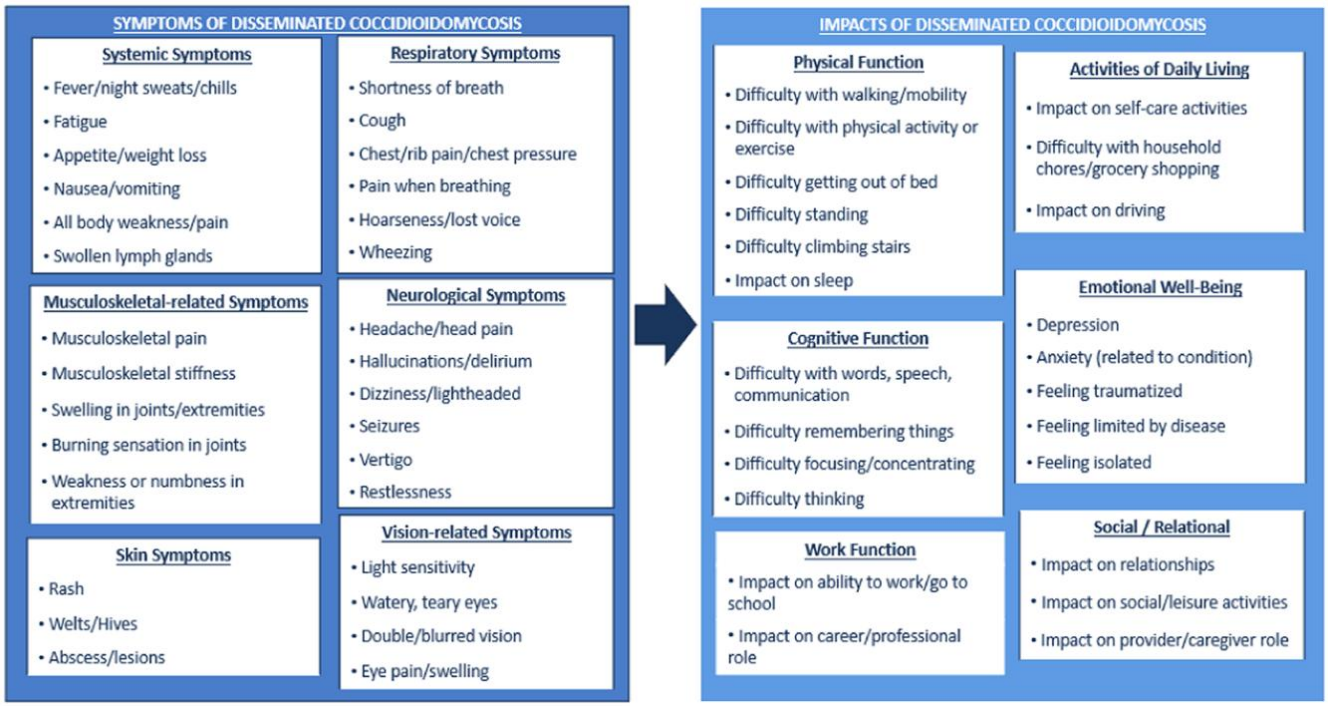
Finalized patient-centred conceptual model of disseminated coccidioidomycosis.

Key symptoms and impacts were identified based on frequency of mentions across subgroups and importance as described by patients. The draft CM was updated with key concepts from the patient-participant and clinician interviews, and insights from a patient advocate living with the condition (Figure 4). Saturation analyses conducted for the subgroups of interest and total Ce patient sample showed saturation of the most relevant concepts.

### Item generation

Nineteen items were drafted across 5 HRQoL impact domains. Item wording incorporated patient language and phrasing identified in the Ce interviews. The response scales and recall period were selected based on patients’ experiences of the concept, alongside PRO regulatory guidance. Meetings were held between the research team, the patient advocate and the bilingual researcher who conducted the Spanish-language interviews, in which initial items were reviewed and revised. The bilingual researcher provided feedback on the translatability and cultural appropriateness of the items for the Spanish-speaking patient population.

### Cognitive debrief findings

The 19 items were generally clear and well-understood by participants. Minor revisions were made to 7 items to reduce item difficulty level (e.g. reducing the duration of walking from 15 to 10 min), and simplifying wording to help increase overall comprehension. Partway through interviewing, a new item assessing ‘napping’ was introduced based on clinician feedback as being a missing concept. This item was well received and reported as important by the remaining 7 participants who were debriefed on this item.

Based on participant feedback, revisions were made to difficulty response scale by removing a problematic option (‘N/A—Not applicable’) and replacing the ‘Unable to do’ response option with ‘Cannot do’. The measure instructions were shortened, and item language throughout the Spanish version was simplified in addition to the overall item/measure edits.

### Item harmonization

Item harmonization meetings were held by the research team to review and agree on edits and finalise the PRO measure; this included meeting with the FDA to receive feedback on the PRO measure. The newly created item, ‘needing to sleep or nap during the day’, was agreed to be important to include in the measure and was added to the physical function domain. The final version of the VF-PRO questionnaire contains 20 items assessing the HRQoL impacts of disseminated coccidioidomycosis. The measure is estimated to take 5–10 min to complete. Table 2 provides examples of the finalized PRO items.

**Table 2.** Example of final PRO items

PRO domain	Concept	Example items
Physical function	Difficulty with walking/mobility	How difficult was it to **walk at a normal speed for 10 min** due to your Valley Fever?
Activities of daily living	Difficulty with household chores, grocery shopping	How difficult was it to **do chores or work around the house** due to your Valley Fever?
Social/role function	Impact on ability to work or go to school	How difficult was it to **work or go to school** due to your Valley Fever?
Cognitive function	Difficulty with words, speech, communication	How often did you have difficulty **finding the right words or phrases** due to your Valley Fever?
Emotional function	Depression	How often did you **feel depressed** due to your Valley Fever?

## Discussion

There is a dearth of research into the impacts of invasive and endemic fungal diseases on the lives of patients living with these diseases.^19^ Generic measures, such as the PROMIS-10, lack the specificity required to measure the impact of a disease such as disseminated coccidioidomycosis. Therefore, the intent of this research was to develop a disease-specific PRO measure for use in future clinical trials and clinical practice. The methodology aligned with US FDA Guidance for Industry and Patient-Focused Drug Development guidances and involved patient and clinician interviews, item generation, CD and instrument harmonization meetings.^8,12^

The Ce interviews captured, for the first time, the experience of living with chronic, disseminated coccidioidomycosis from the patient perspective, including the path to diagnosis, symptoms, and disease impacts on the patient’s daily life. Patients with inadequately controlled infection (i.e.  ≥ 6 months since diagnosis/treatment with ongoing, active symptoms;  ≥ 1 failed azole) were interviewed for this study to reflect the expected clinical trial target patient population for novel treatments for disseminated VF.^8,12^ Patients described widespread and debilitating symptoms, which significantly impacted their quality of life. Unexpectedly, no major differences were found in disease experience when comparing CNS and non-CNS patients. For example, non-CNS patients experienced systemic symptoms such as profound fatigue, and cognitive symptoms like forgetfulness, and difficulty focusing and information processing as frequently as CNS patients. Conversely, neurological symptoms (e.g. head pressure, stroke-related symptoms, paralysis) were only experienced by CNS patients, and balance/instability/falling (ataxia) was reported more by CNS than non-CNS patients (46.0% versus 17.0%). Similarities in symptom experiences between patient groups may be related to triazole treatment side effects, as patients were not always certain of the differences between the two since most patients are unable to stop antifungal treatment once their disease has disseminated. Attribution will be explored during the validation phase of the study.

No major differences were identified in the types of impacts experienced between the CNS and non-CNS groups within the physical, emotional, cognitive and social/role domains. Minor differences were found between English- and Spanish-speaking participant experiences; for example, Spanish-speaking participants discussed impacts to their capacity to work but did not specifically endorse impacts on their professional or career roles. Further, they did not endorse as many emotional functioning concepts (e.g. feeling angry, judged/stigmatized and burdensome). Although differences were noted, it may be speculated that Spanish-speaking patients have greater risk for more severe disease related to limitations in healthcare access and are more likely to hold occupations with multiple risk factors for VF.^20,21^

The research team jointly developed the VF-PRO, which in its preliminary form consisted of 19 items across 5 domains (physical functioning, activities of daily living, social/role function, cognitive function and emotional function), and in its final form consists of 20 items resulting from the addition of an item to the physical functioning domain. The recall period assesses the item concept within the past 7 days and utilizes 2 response scales: difficulty (items 1–14) and frequency (items 15–20).

### Strengths and limitations

Particular attention was given to recruiting a racially, ethnically and clinically diverse sample that reflects the overall disseminated patient population. Outreach through community resources was leveraged to reach recruitment targets for race, primary language, occupation and education.^22,23^ To illustrate, interview samples included ∼15% of patients who did not complete high school, critical group for CD interviews to ensure that the questionnaire is understandable for patients of all education levels. Further, the sample was diverse in terms of dissemination type, ethnicity, employment status and geographic location. The patient feedback collected in this study is thus representative of the wider population of people who have chronic, disseminated coccidioidomycosis.

Despite this considerable strength, it is important to consider limitations to study findings. First, both symptoms and azole side effects were described by patients during interviews as a part of their overall disease experience. While many discussed thoughts on their symptoms due to the infection or treatment, most could not clearly distinguish between the two. Only ‘hair loss’ and ‘dry skin’ were clearly attributed by most patients to their treatment; therefore, these concepts were excluded from the CM. Second, although recall bias can be an issue within qualitative research, the focus of this study was to understand patients’ experience as they live with this chronic condition. To minimize recall bias, during analysis, patients’ descriptions of their most recent or current impact experiences were given greater importance than those that occurred earlier in their disease experience (e.g. during the acute phase of infection).

### Conclusions

The methods used in this qualitative study have resulted in a 20-item *de novo* measure designed to meet current regulatory requirements and guidances. The qualitative evidence supports content saturation and so validity, suggesting that the measure has a comprehensive set of items to describe the impact of disseminated coccidioidomycosis.

Psychometric validation of the VF-PRO is currently underway to assess the psychometric parameters of reliability, validity, sensitivity to change, meaningful change threshold as well as confirm the factor structure and scoring of the measure. Once fully validated, it is anticipated that the VF-PRO can be incorporated into clinical drug development programmes and real-world studies to measure changes in HRQoL in response to novel therapies and in clinical practice to monitor health status and response to treatment in patients with VF.

## Data Availability

The datasets generated and/or analysed during the current study are not publicly available but are available from the corresponding author on reasonable request.
